# Robot End-Effectors Adaptive Design Method Based on Embedding Domain Knowledge into Reinforcement Learning

**DOI:** 10.3390/s26061933

**Published:** 2026-03-19

**Authors:** Yong Zhu, Taihua Zhang, Yao Lu, Liguo Yao

**Affiliations:** 1 School of Mechanical and Electrical Engineering, Guizhou Normal University, Guiyang 550025, China; yzhu@gznu.edu.cn (Y.Z.); yao.lu@gznu.edu.cn (Y.L.); lgyao@gznu.edu.cn (L.Y.); 2 Guizhou Key Laboratory of NewGen Cyberspace Security, Guizhou Normal University, Guiyang 550025, China; 3 Technical Engineering Center of Manufacturing Service and Knowledge Engineering, Guizhou Normal University, Guiyang 550025, China

**Keywords:** adaptive design, robot end-effector, knowledge graph, reinforcement learning, environmental interaction

## Abstract

Existing robot end-effectors design methods lack structured domain prior knowledge support and have insufficient interaction with the environment, making it difficult to guarantee the accuracy of the design results. An adaptive design method is proposed that deeply embeds domain knowledge of end effectors into the design process, treats key design parameters as environmental variables, and optimizes them adaptively through reinforcement learning algorithms in perception and feedback. In a simulation environment constructed by combining a knowledge graph, a two-finger translational gripper is used as an example robot end-effector to acquire target data via sensors, and reinforcement learning is used to adaptively optimize the gripper’s key parameters. Experiments are conducted on a simulation platform with three typical tasks, yielding the optimal parameter range. Compared to the proximal policy optimization (PPO) algorithm, which has no prior knowledge input, the knowledge graph embedding proximal policy optimization (KGPPO) algorithm improves the average reward for gripper length and gripper force by 63.96% and 43.09%, respectively, for grasping eggs. The KGPPO algorithm achieves the highest average reward and the best stability compared with other algorithms. Experiments show that this method can significantly improve the efficiency, stability, and accuracy of design parameter optimization.

## 1. Introduction

Robot end-effectors are the “last mile” for robots to interact with the physical world. They are key components for robots to complete tasks such as grasping, gripping, handling, and assembling [1]. Their performance directly determines whether robots can grasp stably, operate accurately, and interact safely. They are the key link connecting perception, decision-making, and action execution [2]. In recent years, the rapid development of intelligent upgrading of global manufacturing, automation of warehousing and logistics, and emerging scenarios, such as medical rehabilitation and home services, has led to robots moving from structured environments to open, dynamic, and unstructured environments, which puts forward higher requirements for end-effectors: they must not only adapt to multi-shaped objects, but also maintain robustness and reliability under uncertain friction, random disturbance, and task switching conditions. At the same time, the rise of embodied intelligence has made the ability to “learn and complete complex operations in the real world” a research hotspot [3], and robot end-effectors are among the core carriers of embodied intelligence. In reality, problems, such as grasping failure, slippage, and object damage, often stem from mismatches between the end-effectors’ structure and parameters, an unclear contact mechanism, or an inconsistent control strategy and mechanical capability. More importantly, the design of end-effectors is essentially a multi-objective and multi-constraint engineering system problem: it must not only meet the performance indicators, such as grasping stability, force closure, and anti-slip, but also take into account engineering constraints such as safety (damage threshold for vulnerable objects), energy consumption, material and manufacturing costs, and space size [4]. Faced with the high diversity of object attributes and task requirements, the traditional “experience-driven” and “manual parameter tuning” design mode has a long design cycle, relies on expert experience, has high design costs, and is difficult to achieve rapid iteration and cross-scenario reuse; while pure data-driven learning methods often face challenges such as low search efficiency, weak interpretability, and difficulty in guaranteeing engineering feasibility [5]. Therefore, in order to promote the long-term development of embodied intelligence and intelligent manufacturing, it is necessary to develop a method system that can integrate domain knowledge, can perform closed-loop optimization in simulation and real interaction environments, and support the adaptive design of robot end-effectors [6], so that the design process is closer to the real working conditions, can significantly improve optimization efficiency and design result accuracy, and reduce design cycle and trial production costs, which has important theoretical value and urgent engineering significance.

Extensive research has been conducted in the design of robot end-effectors, resulting in multiple technological approaches and some progress. Seo et al. [7] designed a robust and mechanically simple compliant finger gripper that combined the advantages of both force closure and form closure mechanisms to improve grasping performance. The proposed gripper consists of two carbon fiber reinforced plastic plates and operates. Its deformation is induced by linear actuation, allowing it to securely grasp various objects through compliant motion. Due to the limitations of grippers operating in a single rigid or flexible state, Wang et al. [8] designed a reconfigurable gripper that can switch between rigid, rigid bending, and flexible states. They developed mathematical models for the three states, tested the performance of different gripping configurations, and ultimately explored applications of rigid-flexible cooperative operation. Firth et al. [9] used soft robotics technology in an iterative design process to create a hybrid end effector capable of using multiple tools. Zhao et al. [10] proposed an innovative end-effector based on a deployable mechanism, which is an expandable and foldable scissor mechanism that can harvest one or more fruits at the same time. To achieve fast and safe grasping, Zhang et al. [11] proposed a novel two-finger soft gripper design. The two fingers of this gripper are tendon-driven, and two bistable mechanisms are used to achieve rapid response and coordinated bending. The current soft gripper can integrate speed, safety, and compliance through simple mechanical designs. Li et al. [12] developed a rope-driven adaptive end-effector for non-damaging pear picking, drawing on human hand and grasping methods, and robot models. This effectively improved the grasping rate and reduced fruit damage. Lian et al. [13] proposed an adaptive end-effector grasping posture control algorithm and determined the most suitable grasping posture for the manipulator based on posture analysis of the sweet pepper stem. Based on this, they designed a novel end-effector for sweet peppers. Milojević et al. [14] proposed a novel, simple, adaptive, and multifunctional two-finger gripper design method for soft robots based on a flexible mechanism. Prototype testing of the gripper demonstrates that it can reliably, safely, and quickly grasp and manipulate various objects.

At the methodological design level, most scholars have conducted research on introducing knowledge graphs and knowledge reasoning to express design knowledge, as well as on using adaptive algorithms for control and planning. To address the limited usability of soft robotic hands in restricted or cluttered spaces, Bo et al. [15] proposed a method for automatically designing soft-rigid composite spoon-shaped attachments, which served as “embedded constraints.” Given a gripper and a large number of objects, they derive the optimal design parameters for the spoon-shaped attachment for each object by solving an optimization problem. Park et al. [16] developed an intelligent robotic gripper system using 3D object recognition with CAD and point cloud data, reinforcement learning for robotic arms, and custom-printed 3D grippers, enabling manufacturing systems to achieve autonomy, self-identification, and self-adaptation. To enable the robot to maintain the precise position and orientation of the grasped object, Yadav et al. [17] proposed a method for reliable grasping by combining a novel passive gripper design with an advanced adaptive control method. To bridge the gap in research on the synthesis of two-handed grasping in dexterous hand manipulators, Shao et al. [18] proposed the BimanGrasp algorithm for synthesizing two-handed grasping on 3D objects. The algorithm generates grasping poses by optimizing an energy function that considers both grasping stability and feasibility. To design a gripper that can grasp various objects and achieve optimal gripping positions, Yi et al. [19] proposed a co-design framework that generated an optimized soft gripper’s block-wise stiffness distribution and its grasping pose, using a neural physics model trained in simulation. To address the issues of low load capacity, insufficient force sensing, and weak grasping stability in existing grippers, Dong et al. [20] proposed a novel under-actuated gripper featuring two 3-joint fingers driven by a single actuator, achieving sensor-free force feedback through a hierarchical, dual-mode architecture that combines systematic mechanism modeling with a long short-term memory network. For the problem of autonomous control of unmanned flight operations in an unknown disturbance environment, Zhang et al. [21] proposed a knowledge push technology based on quality function knowledge deployment and developed a prototype system of a computer-aided innovative design platform to implement this knowledge push model, and through its application on the design case of a space robot gripper, the practicability and validity of the prototype system were demonstrated. Beddow et al. [22] combined force feedback with reinforcement learning to design a three-degree-of-freedom caging-inspired gripper that can grasp by trapping objects with three compliant fingers and a movable palm.

In summary, the robot end-effectors have accumulated research results in mechanism design, algorithm optimization, and product design methods. However, there is a lack of researchers focusing on the systematic organization of knowledge and the expression of computable constraints for robot end-effectors, as well as on the interaction and perception between robot parameter design and the robot’s working environment and specific tasks, to design optimal parameter values.

**Figure 1:**
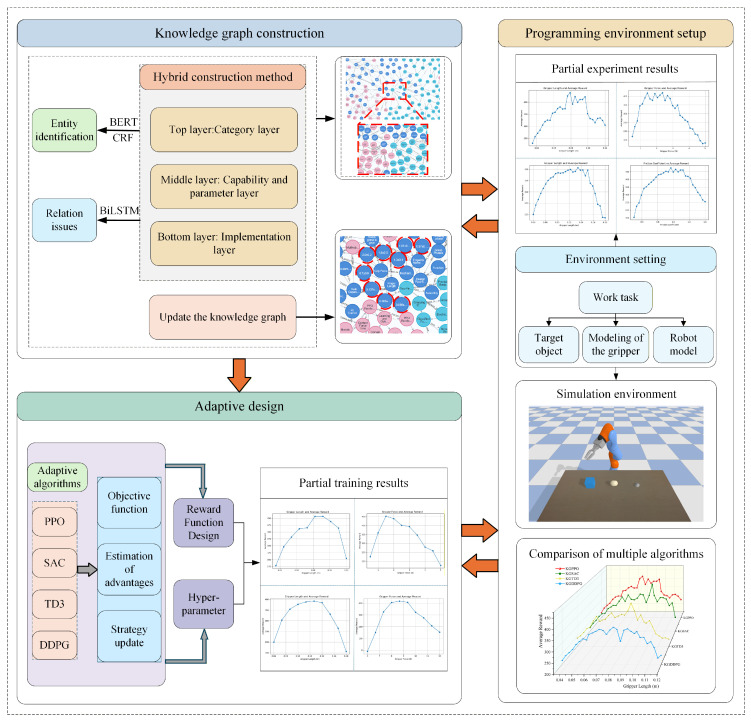
Roadmap of adaptive design technology for robot end-effectors.

The technical approach of this paper is shown in Figure 1. The main research contents are as follows. A dedicated knowledge graph for robot end-effectors design is constructed and deeply embedded into the design process. It integrates domain principles, case studies, and rules to form a comprehensive knowledge base for design. A named entity recognition method that integrates semantic representation learning and conditional dependency modeling accurately identifies domain entities and solves the problem of ambiguous relation representation through a context-aware mechanism. A complete “perception-decision-learning” adaptive design closed loop is designed and validated. During reinforcement learning in a simulation validation environment, design parameters are iteratively optimized using a reward function. This closed loop ensures that the system’s design capabilities continuously grow with experience, possess lifelong learning characteristics, and provide a sustainable, scalable solution for addressing the challenges of “small batch, multi-variety” production. A novel adaptive design paradigm for robot end-effectors that integrates knowledge graphs and reinforcement learning is proposed. A self-built simulation environment is constructed, incorporating the knowledge graph and adaptive design, to create a closed-loop design ecosystem. This ecosystem combines systematic domain knowledge with autonomous optimization algorithms. The optimized design and its performance metrics are fed back into the knowledge graph as a new “design experience,” dynamically updating it.

The remainder of this paper is organized as follows. Section 2 defines the operational tasks, constructs the robot end-effectors model, and builds the simulation environment. Section 3 reviews and organizes professional knowledge in the field of robot end-effectors, constructs a knowledge graph structure framework for robot end-effectors, designs adaptive algorithms, and trains them. Section 4 compares the impact of domain knowledge guidance on the PPO algorithm’s results. Using the knowledge graph as prior guidance, experiments are conducted on three work tasks using the KGPPO algorithm, drawing conclusions and comparing multiple algorithms. Section 5 summarizes the research results and provides an outlook on future research.

## 2. Experimental Tasks Setting and Environment Modeling

### 2.1. Operational Tasks and Parameterized Models

#### 2.1.1. Operational Tasks

To systematically evaluate the grasping, handling, and placement capabilities of an end-effector under different object properties and contact constraints, this paper designs three representative operational tasks. All three tasks follow a unified sequence of actions: the end-effector first achieves stable gripping of the target object within an initial area, then performs a vertical lifting motion of 10 cm, followed by a horizontal displacement of 10 cm along the robot’s front direction, ultimately achieving stable placement with low impact and controllable posture. Successful completion of the task requires not only no significant slippage, rotational instability, or detachment of the object during handling, but also controlled contact impact during placement to avoid secondary damage or posture deviation.

Task 1 (Egg Grabbing and Gentle Placement): Eggs are typically fragile targets, emphasizing control of the upper limit of applied force and the distribution of contact pressure while meeting anti-slip constraints to avoid damage from localized stress concentration. This task places higher demands on gripper force adjustment, closing speed control, and contact stability.

Task 2 (Cube Grabbing and Precise Transport): Cubes have regular geometry and high rigidity, resulting in a relatively low risk of damage. However, the evaluation focus shifts to operational accuracy and stability. This task better reflects the strategy’s ability to optimize pose control, force-displacement coordination, and end-effectors trajectory smoothness.

Task 3 (Gear Grabbing and Geometric Constraint Adaptation): Gears have irregular shapes and abrupt local geometric changes in their tooth profiles, resulting in a limited gripping area and a tendency to deflect. This task requires a strategy capable of selecting more appropriate gripping points and maintaining stable contact under complex geometric conditions.

To improve the generalization ability of reinforcement learning training and avoid overfitting of the policy to a single initial condition, this paper randomizes the key initial states during the environment reset and iterative training process, including the initial position and orientation of the target object on the workbench, which are randomly sampled within a preset range.

#### 2.1.2. Parameterized Model

In the design process of robot end-effectors, there is a strong coupling among structural dimensions, driving capability, contact characteristics, and control strategy [23]. To achieve task-oriented, computable design and adaptive optimization, this paper parametrically models the key design elements of the end-effectors, with the main parameters shown in Table 1.

### 2.2. Selection and Design of End-Effectors

A robot end-effector is a device installed at the very end of a robot arm. It is the component that directly interfaces with the robot’s environment or workpiece and performs tasks. Robot end-effectors are designed for contact and force transmission and need to achieve stable, reliable, and safe operation under complex geometric constraints, frictional uncertainties, and dynamic disturbances. Their core task is to complete specific operations.

Among the many types of end-effectors, the two-finger translational gripper is widely used in typical tasks, such as sorting, loading and unloading, assembly, and handling, due to its compact structure, clear control interface, and strong practicality [24]. This type of gripper achieves grasping by moving two fingers synchronously in opposite directions, providing reasonable normal constraints on the grasped object, having frictional anti-slip capabilities, and possessing good versatility and maintainability. This feature of “simple structure but good contact and force control” allows it to cover the gripping needs of regular objects (such as cubes) and semi-regular objects (such as gears), and can grasp fragile objects (such as eggs) under reasonable force control and contact, and can complete the set operational tasks with high reliability. Therefore, this paper selects the two-finger translational gripper as the research object.

**Figure 2:**
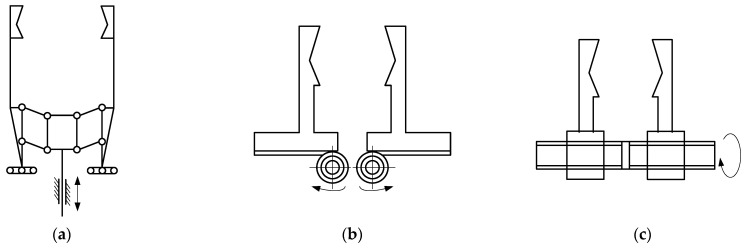
Translational-type grippers. (**a**) A gripper that uses linkages and guide grooves to drive the finger translation; (**b**) a gripper that uses gears and racks to drive the finger translation; (**c**) a gripper that uses bidirectional screws to drive the finger translation.

The two-finger translational gripper structure is shown in Figure 2. When the gripper clamps and releases the workpiece, the fingers move in translation while keeping the clamping center fixed and unaffected by changes in the workpiece diameter. The gripper shown in Figure 2a uses linkages and guide grooves to drive the fingers’ translational movement while maintaining a constant clamping center position; this type of gripper is also called a concentric gripping mechanism. The gripper shown in Figure 2b uses gears and racks to drive the finger translation. The gripper shown in Figure 2c uses bidirectional screws to drive the finger translation. In the task described in this paper, the target object to be gripped contains fragile objects such as eggs. The bidirectional screw offers a wide, controllable gripper force range, strong gripping stability, and more stable force control to prevent dropping. Therefore, the two-finger translational gripper shown in Figure 2c, which uses a bidirectional screw to drive the finger translation, was selected.

The gripper design process involves considerations such as the feasible design variable domain and the most common failure modes in the grasping task. The main theoretical derivations are as follows, and all of these theories are stored in a knowledge graph.

1. Strength and Stiffness of Finger Structure

The length of the fingers has a certain influence on the strength and rigidity of the grippers. If we approximate the finger as a cantilever beam, the maximum bending stress is shown in Equation (1).

(1) $$\sigma_{max}=\frac{M_{max}c}{I}=\frac{FL(h/2)}{\left(bh^{3}/12\right)}=\frac{6FL}{bh^{2}},\sigma_{max}\leq \sigma_{allow}$$

Equivalent stiffness is shown in Equation (2)

(2) $$k=\frac{F}{\delta }=\frac{F}{\left(\frac{Fl^{3}}{3EI}\right)}=\frac{3EI}{L^{3}},\delta \leq \delta_{allow}$$

where *b* is the width of the rectangular cross-section, *h* is the thickness, *L* is the length, *F* is the equal transverse force on the end, *I* is the moment of inertia of the cross-section, *M* is the bending moment, and *E* is the modulus of elasticity. Therefore, the longer the fingers, the bigger the bending stress and deformation, and the lower the stability and control precision of the grasp.

2. Task Load and Gripper Force

When the vertical upward acceleration of the gripper is $a_{z}$, the gripper force is shown in Inequality (3).

(3) $$2\mu F_{n}\geq m(g+a_{z})\Rightarrow F_{n}\geq \frac{m(g+a_{z})}{2\mu },F\geq s\cdot \frac{m(g+a_{z})}{\mu }$$

where the safety factor *s* > 1, *m* is the mass of the object to be grasped, *F_n_* is the normal force, and the gripper force *F* = 2*F_n_*. The gripper force of the gripper is related to the mass, acceleration, and coefficient of friction of the object to be gripped, as described above. Therefore, a constraint on the relationship between the gripper force and the friction coefficient is imposed in the parameter optimization process described later.

3. Anti-overturning Moment

The maximum anti-overturning torque provided by friction, as shown in Equation (4).

(4) $$M_{\max}\approx \sum_{i=1}^{2}\mu F_{n,i}r_{i}\geq M_{ext}$$

where is the overturning moment generated by the external force about the contact point, *r_i_* is the lever arm of the frictional force about the overturning axis.

4. Contact Stress

Using the Hertzian sphere-plane equivalent contact model, the peak pressure is shown in Equation (5).

(5) $$p_{0}=\frac{3F_{n}}{2\pi a^{2}}=\frac{3F_{n}}{2\pi {\left(\frac{3F_{n}R}{4E^{*}}\right)}^{2/3}}=\frac{{\left(6F_{n}\right)}^{1/3}{\left(E^{*}\right)}^{2/3}}{\pi R^{2/3}}\leq p_{0,allow}$$

where *R* is the equivalent radius, $E^{*}$ is the equivalent modulus, *F_n_* is the normal force, and a is the contact radius.

The parameter design in this paper focuses on key parameters, such as gripper length, gripper force, friction coefficient, and stroke, after interaction with the environment. The two-finger translational gripper consists of a fixed base and two symmetrically arranged fingers, each finger being connected to the base via a single-degree-of-freedom movable joint. The three-dimensional geometric model of the gripper was created in SolidWorks 2023 software, and the mass and moment of inertia of each rigid body were estimated based on the mass distribution. Subsequently, the geometric model was converted into a URDF file according to the mechanical structure. In the URDF, information, such as rigid bodies, joints, visual meshes, and collision meshes, was defined sequentially, while a suitable friction coefficient was set for the fingertip contact surface. Finally, the loadURDF format file of the self-built gripper model was imported into the PyBullet simulation environment.

### 2.3. Simulation Environment Construction

This paper presents a robot grasping simulation platform built upon the open-source physics simulation engine PyBullet. PyBullet provides functions for rigid-body dynamics simulation, collision detection, joint control, and camera rendering, and can easily load URDF-format robot and environment models, making it suitable for research on robot motion planning and reinforcement learning. This study was developed using Python 3.11.7 and PyBullet 3.2.7 on Windows, with a simulation step size of 1/240 s and a gravitational acceleration of 9.81 m/s^2^.

Regarding the robot body, this paper selects the KUKA LBR iiwa 7 R800 seven-DOF industrial robot (Augsburg, Germany) provided in the PyBullet library. This robot model features a compact structure and a large workspace, with a standard installation interface at the end-effector flange for easy assembly of various end-effectors. By calling the loadURDF interface to load the official URDF file and setting appropriate base positions and orientations, a virtual model consistent with the actual industrial robot structure can be established in the simulation environment. Subsequently, by traversing the joint indexes, the link numbers of the end-effectors are determined, providing a coordinate system reference for the subsequent installation of the self-built gripper.

To simulate the grasping task, this paper constructs three target objects: an egg, a cube, and a gear. The egg is approximated as an ellipsoid with major and minor axes of 0.055 m and 0.042 m, respectively, and a mass of 0.054 kg; the cube is modeled as a rigid body made of aluminum alloy with a side length of 0.08 m and a mass of 1.5 kg; the gear has a tooth tip circle diameter of 0.04 m, a tooth height of 0.0025 m, a tooth width of 0.02 m, is made of 45 steel, and has a mass of 0.2 kg.

**Figure 3:**
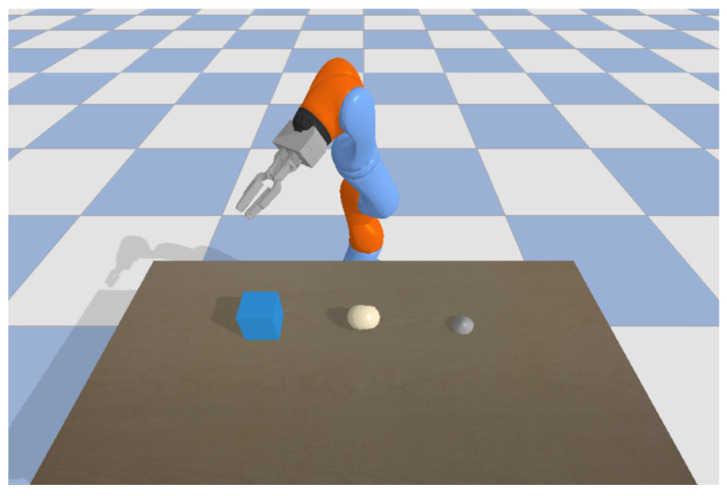
KUKA LBR iiwa 7 R800 robot assembling a self-built gripper to grasp an object.

The assembly of the KUKA LBR iiwa 7 R800 robot with its self-built gripper was achieved through coordinate transformation and constraint relationships. A simulation environment was built in PyBullet, and egg, cube, and gear models were imported into the environment. All objects were assigned reasonable density and friction parameters and placed on a table within the workspace, as shown in Figure 3. This environment defines a continuous motion space and an observation space containing 15-dimensional information, including the robotic arm’s state, changes in finger travel, object pose, and finger contact state.

At the start of each simulation, the robot and gripper are initialized to a given safe posture, with the target object randomly placed within a specified range. Then, the robotic arm’s end-effector moves to a pre-grasping pose above the object according to a planned trajectory, and the gripper gradually closes until its two fingers make stable contact with the object’s surface. After gripping, the robotic arm raises its end-effector vertically. If the object is successfully lifted off the table to a predetermined height and moved to a designated position without slipping within several simulation steps, the gripping is considered successful. Based on this simulation platform, subsequent chapters will introduce adaptive algorithms to optimize various gripper design parameters.

## 3. Adaptive Design Frameworks for Robot End-Effectors

### 3.1. Domain Knowledge Modeling for Robot End-Effectors

Specialized knowledge of robot end-effectors is a crucial resource. In the process of designing robot end-effectors, it is difficult to systematically express the coupling relationships and uncertainties among structure, materials, control, and tasks based on traditional experience, and it is impossible to accurately quantify information entropy during design. Effective methods for storing, organizing, and managing this knowledge enable the accumulation, transfer, and reuse of design experience. This assists researchers in quickly comparing solutions, analyzing performance, and optimizing parameters, thereby improving design efficiency and grasping reliability. A knowledge graph is an efficient knowledge management tool [25] that consists of entities, their relationships, and attributes. It can associate end-effector types, functional requirements, key parameters, experimental data, failure modes, and other multi-source knowledge in a structured way, providing new technical support for the construction of an end-effector design knowledge base and for intelligent auxiliary design [26]. The construction technology of the knowledge graph is mainly divided into three types: bottom-up, top-down, and hybrid [27]. This paper aims to determine the overall framework of the robot end-effectors from top to bottom, select specific end-effectors based on the requirements of the operational task, and fill in the data and knowledge from various fields from bottom to top. Therefore, a hybrid construction method is adopted to model the robot end-effectors field.

#### 3.1.1. Hierarchical Modeling Approach and Knowledge Scope Definition

This paper adopts a three-layer modeling framework: “category layer, capability and parameter layer, implementation layer” to construct a knowledge graph. The types and instances in the top layer determine the range of capabilities and key parameters in the middle layer. The capabilities and parameters in the middle layer, in turn, rely on the underlying control algorithms, mechanical mechanisms, and data-driven strategies for implementation and verification. This framework decouples structural information, capability requirements, and implementation mechanisms while preserving cross-layer relationships to enhance the scalability and reasonability of knowledge.

1. Top Layer: Category Layer

The top layer is used to characterize the “category-instance” system of robot end-effectors, which is the main organizational backbone of the Atlas. There is a wide variety of robot end-effectors. We will classify robot end-effectors according to their functions, task requirements, driving methods, flexibility and complexity, structure and form, and application fields [28]. Grasping end-effectors are used to grasp and move objects; manipulating end-effectors are used to complete specific tasks or operations; sensing end-effectors are equipped with sensors and can obtain feedback information from interaction with the environment; multi-functional end-effectors can perform multiple tasks or functions at the same time. In the classification by driving method, the electric drive type uses an electric motor as the driving source, providing high-precision control and fast response; the pneumatic drive type drives the actuator via compressed air; the hydraulic drive type provides high power via a hydraulic system. In the classification by structure and form, rigid end-effectors have a rigid structure and are usually used for tasks that require precise control and rigid grasping; flexible end-effectors have a soft structure and can adapt to objects of various shapes; and flexible, versatile end-effectors can assume multiple forms through multiple degrees of freedom and joints. In terms of application areas, industrial robot end-effectors are used in automated production lines; medical robot end-effectors require extremely high precision and reliability; service robot end-effectors include home service robots and restaurant delivery robots; and agricultural robot end-effectors include harvesting robots. In terms of flexibility and complexity, fixed end-effectors remain stationary and are typically used for simple tasks; variable end-effectors have multiple degrees of freedom and can perform complex tasks.

2. Middle Layer: Capability and Parameter Layer.

The middle layer expresses “what the end-effectors can do and what is adjustable in design,” serving as a crucial layer for design decisions and task matching. This study categorizes the middle layer modeling into four core concepts: function, design parameters, control methods, and task capabilities. A unified relational schema connects these to the top-level entities and the bottom-level implementation. End-effector functions are categorized and described based on “what it does,” refined to the granularity of mappable tasks to ensure stable alignment between functional nodes, task requirements, and evaluation metrics. Key parameters can be summarized into geometric, mechanical, execution, energy consumption, and safety and reliability parameters. Control methods describe the “control paradigm” rather than specific algorithm details, reflecting the matching relationship between control strategies, structure, and task requirements. Common control methods include position control, speed control, force control, impedance control, and learning-based strategy control. Task capabilities characterize “to what extent it can be done,” forming the core of task matching and recommendation. Capability descriptions are primarily expressed through metrics such as object adaptability, grasping performance indicators, fine manipulation capabilities, and safety indicators.

3. Bottom Layer: Concrete Implementation Layer

The bottom layer is used to characterize the specific implementation mechanisms and evidence sources of the capabilities. This layer defines four node types: specific control algorithms, kinematic modeling, sensor data, and learning optimization strategies. These nodes form a reasonable link through explicit dependencies, enabling the specifications described in the middle layer to be verified, reproduced, and iterated. Specific control algorithms further refine the middle-layer “control methods” into implementable algorithmic units, including classical control, force-dependent control, optimization and predictive control, grasping planning, and learning algorithms. Kinematic modeling translates concepts such as “grasp stability, slippage, and damage” to a computational level. The bottom-layer model nodes describe key mechanisms such as kinematic constraints, contact mechanics, and friction laws, and object properties and damage thresholds. Sensor data is the explicit modeling of sensors and data at the bottom layer, supporting the verifiability of “perception-supported control.” Learning and optimization strategies meet the iterative needs of end-effectors design and control.

#### 3.1.2. Relationship Types in the Field of Robot End-Effectors

**Figure 4:**
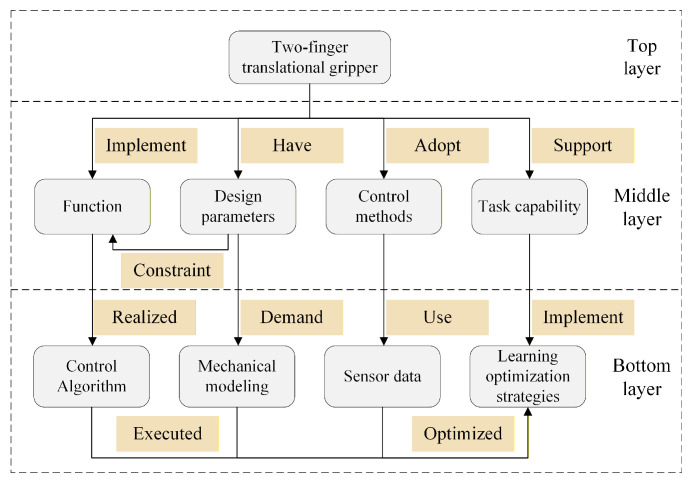
Relationships between entities in a two-finger translational gripper.

The top layer of the knowledge graph of robot end-effectors includes the basic categories of end-effectors. Different categories are parallel to each other, and there is an inclusion relationship between the same category [29]. For example, the two-finger translational gripper, the relationship between the levels is shown in Figure 4.

#### 3.1.3. Data Sources

Constructing a knowledge graph for robot end-effectors requires higher-accuracy, fine-grained data derived primarily from reference books, research materials, and professional literature in the field [30,31].

### 3.2. Steps for Constructing a Knowledge Graph in the Field of Robot End-Effectors

#### 3.2.1. Data Preprocessing

The data extracted in this paper primarily consisted of books, research materials, and the literature on robot end-effectors. After converting these materials into a unified text format, they needed to be preprocessed, primarily including text cleaning and the deletion of stop words, to facilitate subsequent extraction work [32]. Text cleaning is a key step in data preprocessing. First, the collected books, research materials, literature, and standard documents are converted into a unified text format. For books, non-textual content, such as the cover, table of contents, and acknowledgments, is removed, leaving only the main text. For materials and literature, auxiliary content, such as cover information, abstracts, and references, is removed, leaving only the core data and analysis. For standard documents, information such as the standard number, issuing organization, and publication date on the first page is removed, and the preface, table of contents, and supplementary content (e.g., figures and tables) in the appendix are also removed, leaving only the core text content.

#### 3.2.2. Named Entity Recognition Based on BERT + CRF

This paper employs a named entity recognition model based on Bidirectional Encoder Representations from Transformers (BERT) and Conditional Random Field (CRF). This model fully leverages BERT’s strength in understanding textual context and uses CRF for sequence labeling, effectively achieving named entity recognition in the robot end-effector domain. The application process of the BERT + CRF model in robot end-effector domain entity recognition will be detailed below.

1. BERT Model Application

Unlike traditional one-way models, BERT uses bidirectional encoding to accurately identify entity types in text.

Jieba (a Python library for text segmentation) is used for word segmentation to obtain a word sequence. The input sequence of the BERT model is a sequence $X=\left(x_{1},x_{2},\cdots ,x_{n}\right)$ containing *n* words, where each *x_i_* is an input word. The model encodes the input sequence through a bidirectional Transformer architecture. For each BERT, a context-sensitive vector representation $H=\left(h_{1},h_{2},\cdots ,h_{n}\right),h_{i}\in R^{P}$ is generated, where ***H*** is the hidden layer output computed by BERT, a vector sequence corresponding to each word in the input sequence; *h_i_* is the context-sensitive representation vector of the input word *x_i_* after BERT encoding, containing the semantic information of the word; *P* is the dimension of the BERT hidden layer, and vector ***H*** is passed to the subsequent CRF layer.

2. CRF Layer Model Application

In entity recognition of the knowledge graph of robot end-effectors, there are dependencies between entities, and functional entities are related to control algorithms. Knowledge Rendering Function (CRF) models these dependencies using transition matrices, thereby optimizing label prediction. CRF can select the optimal label sequence based on the label scores output by BERT and the transition relationships between labels.

To capture label dependencies, the CRF layer receives BERT’s output ***H***. The output of BERT is a “semantic vector”, but it cannot be used directly for prediction. A linear transformation layer is needed to obtain a “score for each label.” Mapping ***H*** to a label space, assuming the label set is *k*, the linear transformation is shown in Equation (6).

(6) $$e_{i}=Wh_{i}+b\in R^{k}$$

where *e_i_* is the score of each label assigned to the *i*-th character, ***W*** is the weight matrix of the linear layer, *h_i_* is the BERT representation vector of the *i*-th character, and *b* is the bias term.

Let *A* ∈ *R*^(*K*+2)(*K*+2)^ be the transition matrix between labels. The goal of a CRF layer is to compute the probability of maximizing the correct label sequence *y* given an input ***H***. For an input sequence $H=\left(h_{1},h_{2},\cdots ,h_{n}\right)$ and a label sequence $y=\left(y_{1},y_{2},\cdots ,y_{n}\right)$, the score function of the CRF is shown in Equation (7).

(7) $$s(H,y)=\sum_{i=1}^{n-1}A_{y_{i},y_{i+1}}+\sum_{i=1}^{n}e_{i}(y_{i})$$

where $A_{y_{i},y_{i+1}}$ is the transition score from label *y_i_* to *y*_*i*+1_, representing the dependency relationship between labels; $e_{i}\left(y_{i}\right)$ is the label score output by BERT, representing the score of word *x_i_* belonging to label *y_i_*.

#### 3.2.3. Inter-Entity Relation Extraction Based on BiLSTM-Attention

**Figure 5:**
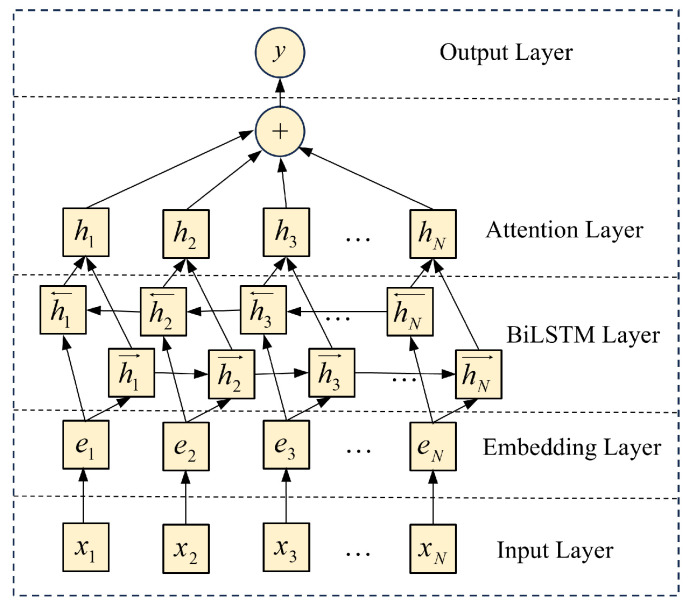
BiLSTM-Attention model architecture.

This paper uses the BiLSTM-Attention model to solve the relation extraction problem. BiLSTM can model the input sequence in both forward and backward directions simultaneously, capturing contextual information in the text, especially long-distance dependencies. The model consists of five layers: an input layer, an embedding layer, an LSTM layer, an attention layer, and an output layer, as shown in Figure 5.

1. Define Relation Recognition Task Formula Symbols

The entity relation triple is π=(h;r;t)|h,t∈E;r∈R, where *h* is the head entity, *t* is the tail entity, *r* is the relationship between entities, *E* and *R* are the entity set and relation set, respectively; the triple *π* represents the entity pair (*h*; *t*) and the relationship *r* between them; the sentence is set as $S=\{w_{1},w_{2},\cdots ,w_{n}\}$; *w_i_* is the *i*-th word in the sentence [33]. For example, in the robot end-effectors knowledge graph, *S* = {“Design Parameters”, “Contains”, “Material”}. The triple is $\pi =(e_{1},r,e_{2})$, where *e*_1_ and *e*_2_ are “Design Parameters” and “Material,” respectively, and “Contains” is the relationship between them.

2. BiLSTM Layer-based Design

In the design of robot end-effectors, describing certain design parameters may involve multiple steps or long causal chains. BiLSTM can help the model better understand the long-distance relationship between a specific parameter and the actual effect.

Given a sentence $S=\{w_{1},w_{2},\cdots ,w_{l}\}$ of length *l*, the representation vector of the *i*-th word is $z_{i}=[w_{wi_{i}};w_{p_{i}};w_{c_{i}}]$, where $w_{wi_{i}}\in R^{d_{w}}$ is a randomly initialized word embedding; $w_{p_{i}}\in R^{d_{pos}}$ is a part-of-speech (POS) embedding, representing character-based word features [34]. Character-level word features are extracted from the character sequence of *w_i_*, and a BiLSTM is then used to capture word dependencies. The input to the BiLSTM network $w_{c_{i}}\in R^{d_{c}}$ is the word vector representation sequence $\left\{z_{1},z_{2},\cdots ,z_{l}\right\}$. The forward and backward LSTM hidden states *h_i_* are concatenated to form the context word vector, as shown in Equation (8).

(8) $$h_{i}=[\vec{LSTM}(z_{i});\overset{\leftarrow }{LSTM}(z_{i})],i\in [1,l]$$

where $h_{i}\in R^{2d_{he}}$, *d_he_* is the dimension of the BiLSTM hidden state.

3. Design of Relationship-Based Attention Mechanism

Words in a sentence have different weights under different relations. To address this, a relation-based attention mechanism is introduced to assign different weights to context words under each relation [35]. This relationship-based attention allocation enables the model to automatically identify the most relevant words in the context of different relationships, thereby improving the accuracy of entity recognition and relationship extraction. The attention score is calculated and shown in Equations (9)–(11)

(9) $$s_{g}=avg\{h_{1},h_{2},\cdots ,h_{l}\}$$

(10) $$e_{ik}=v^{T}\tanh(W_{r}r_{k}+W_{g}s_{g}+W_{h}h_{i})$$

(11) $$\alpha_{ik}=\frac{\exp(e_{ik})}{\sum_{j=1}^{l}\exp(e_{jk})}$$

where $r_{k}\in R^{d_{r}}$ is the trainable embedding of the *k*-th relation, $v\in R^{d_{at}}$, $W_{r}\in R^{d_{att}\times d_{r}}$, $W_{g}$, $W_{h}\in R^{d_{att}\times d_{he}}$ are trainable parameters, and *s_g_* is the global representation of the sentence. In this way, the attention score can measure not only the importance of each word to the relation representation, but also its contribution to the entire sentence. The specific sentence representation is generated by weighting and summing the words in the sentence, as shown in Equation (12).

(12) $$s_{k}=\sum_{i=1}^{n}\alpha_{ik}h_{i}$$

4. Relationship Gating Mechanism

The main function of relation gating is to control the influence of different relations on sentence representation [36]. To adaptively control the relation information from the previous attention layer, a gating mechanism is introduced as a bridge. For the *k*-th relation, the gating operation is defined as shown in Equations (13) and (14).

(13) $$g_{k}=\sigma ((W_{1}s_{g}+b_{1})⊕(W_{2}s_{k}+b_{2}))$$

(14) $$u_{k}=g_{k}\tan h(W_{3}s_{k}+b_{3})$$

where $W_{1},W_{2},W_{3}\in R^{d_{g}\times 2d_{he}}$ and $b_{1},b_{2},b_{3}\in r^{d_{g}}$ are parameters, ⊕ is the concatenation operation, · is the dot product, σ is the element-wise sigmoid activation function, with a return value between 0 and 1, and the result can be regarded as the percentage of information retained. *g_k_* is used to measure the weight of the original sentence representation vector *s_g_* or the relation representation vector *s_k_* for entity extraction.

Using *u_k_* as a preserved relational feature, concatenating *h_i_* and *u_k_* yields the final representation of the *i*-th word, as shown in Equation (15).

(15) $$h_{ik}=h_{i}⊕u_{k}$$

where $h_{ik}\in R^{2d_{he}+d_{g}}$, sentence *S* is now represented as $S_{k}=\{h_{1k},h_{2k},\cdots ,h_{nk}\}$.

#### 3.2.4. Knowledge Extraction Performance Evaluation

To evaluate the reliability of the knowledge extraction process used to construct the robot end-effector knowledge graph, a quantitative assessment was conducted using commonly used information extraction metrics, including precision, recall, and F1-score. The data from Section 3.1.3 is labeled to create a dataset. The labeled dataset includes entities such as categories, structures, functions, parameters, and operational constraints, along with the relationships among them.

Precision measures the proportion of correctly identified entities or relations among all extracted results, while recall represents the proportion of correctly identified entities or relations relative to the ground-truth annotations. The F1-score is the harmonic mean of precision and recall and provides a balanced evaluation of extraction performance.

The mean evaluation results are summarized in Table 2. For entity extraction, the proposed framework achieved a precision of 0.91, a recall of 0.88, and an F1-score of 0.89. For relation extraction, the precision reached 0.88, the recall 0.85, and the F1-score 0.86. These results demonstrate that the proposed knowledge extraction approach can effectively identify domain-specific entities and relationships, providing a reliable knowledge base for subsequent knowledge graph construction and design optimization.

### 3.3. Adaptive Design

Adaptive design is a design philosophy in which a product or system can automatically adjust its structural parameters, control parameters, or working strategies based on perceived information and performance feedback when faced with differences in objects, environmental disturbances, and task changes, in order to maintain or improve target performance [37]. Adaptive design emphasizes incorporating uncertainty into the design loop: the system not only selects parameters in the design phase, but also continuously learns the mapping relationship between “parameters and performance” during operation or iteration, thereby achieving robust adaptation to multiple scenarios [38]. For example, robot end-effectors vary significantly in shape, size, mass, and surface friction. If a fixed finger length, gripper force, or control mode is used, it is often difficult to simultaneously ensure grasping stability and safety. Adaptive design, through mechanical modeling and data-driven optimization, dynamically adjusts the gripper force, opening and closing stroke, impedance parameters, and even adjustable structural components, enabling the system to achieve a balance among multiple objectives such as “anti-slip, anti-damage, energy consumption, and efficiency.”

#### PPO Algorithm

The Proximal Policy Optimization (PPO) algorithm is chosen as the adaptive algorithm [39] because it offers stability and exploration, and it is a reinforcement learning algorithm [40]. The goal of the PPO algorithm is to maximize the long-term reward by optimizing the policy. The key to this algorithm is to limit the magnitude of each policy update to avoid training instability.

1. Policy Objective Function

The PPO algorithm is a widely used policy-gradient-based reinforcement learning method. It adjusts the policy by optimizing an objective function to better adapt to the environment and maximize long-term rewards. Its core idea is to limit the magnitude of each policy update to prevent excessively large updates from causing training instability. The PPO algorithm introduces a trimming objective function to limit the magnitude of each update and avoid over-updating.

The policy objective function of the PPO algorithm is shown in Equation (16).

(16) $$L^{CLIP}(\theta )=E_{t}[\min(r_{t}(\theta ){\hat{A}}_{t},clip(r_{t}(\theta ),1-\varepsilon ,1+\varepsilon ){\hat{A}}_{t})]$$

where r(θ) is the probability ratio between the current policy and the old policy, as shown in Equation (17).

(17) $$r_{t}(\theta )=\frac{\pi_{\theta }(a_{t}\left|s_{t}\right.)}{\pi_{\theta old}(a_{t}\left|s_{t}\right.)}$$

where $\pi_{\theta }(a_{t}\left|s_{t}\right.)$ is the probability that the policy chooses action in state $s_{t}$. ${\hat{A}}_{t}$ is the advantage function, representing the advantage of the current action relative to the average action. It can be calculated using the Generalized Advantage Estimation (GAE) method. ε is a hyperparameter that controls the magnitude of each policy update. $clip(r_{t}(\theta ),1-\varepsilon ,1+\varepsilon )$ indicates a limit on the range of updates to the probability ratio to avoid excessive policy changes.

2. Advantage Estimation

The purpose of the advantage function (${\hat{A}}_{t}$) is to measure the merit of a given action relative to the current policy. Typically, the advantage function of the PPO algorithm is estimated using the Temporal Difference (TD) error, as shown in Equation (18).

(18) $${\hat{A}}_{t}=\sum_{l=0}^{\infty }\left(\gamma^{l}\delta_{t+l}\right)$$

3. Strategy Update

In the PPO algorithm, the policy is updated through small-step optimization. The policy is updated multiple times per iteration, and each update is limited in magnitude to maintain the stability of the training process. The PPO algorithm updates the policy parameters by maximizing the objective function described above.

### 3.4. Reward Function Design

Designing the reward function is a crucial step in the reinforcement learning process, and this design relies on the knowledge graph of robot end-effectors. The design of the reward function has a significant impact on the output results [41]. Rewards can be divided into fixed rewards and variable rewards. The reward function in the experiment comprises multiple components. It is necessary to consult the robot end-effectors’ knowledge graph and consider various factors when the robot grasps. We set the reward value for completing a certain action based on its importance. After completing a grasping task, the rewards are summed to obtain a single reward. Considering the randomness of reinforcement learning in grasping tasks in a simulation environment, after completing a set of iterations with the same parameter settings, we treat the sum of rewards from 200 repetitions of the grasping task as one episode reward, and the average reward over 10 episodes is used as the final output.

#### 3.4.1. Fixed Rewards

The robot receives a fixed reward during the grasping process, which mainly includes the following.

Geometric feasibility reward: Based on the knowledge graph data sources of robot end-effectors, the design of the two-finger translational gripper length *L* and stroke (the distance between the fingers) *S* satisfy Equation (19).

(19) S=2[0.5L−(X+s)]

where *X* represents the finger width, and *s* represents the safe distance between the finger and the end of the stroke.

Therefore, there is a functional relationship between finger length and stroke. When the stroke exceeds the target object, i.e., when the finger length exceeds a certain value, the gripper can wrap the object, satisfy geometric feasibility, and be given a reward; otherwise, it is punished.

Contact reward: The robot needs to ensure that two fingers are in contact with the target object simultaneously to receive a reward. The position and orientation of the target object are obtained from sensors, as is whether the gripper is in contact with it. Simultaneously, the “contact_stable_steps” instruction is used to determine whether the contact is stable.

Alignment reward: A reward is given when the robot’s gripper aligns with the target object.

Lifting reward: When the robot successfully lifts the target object to a threshold, it receives a lifting reward.

Placement reward: The robot rewards placing an object in a designated location.

The reward values are shown in Table 3.

#### 3.4.2. Variable Rewards

The magnitude of the gripper’s movements and the values of its design parameters also need to be reasonable to prevent the gripper from blindly setting unreasonable parameters to maximize fixation. For example, blindly increasing the gripper length to achieve geometric feasibility and gain rewards for lifting. Therefore, it is necessary to adjust the reward control parameters. These mainly include the following:

Action cost: Excessive movements by the robot’s gripper will be penalized.

The design reward for parameters is as follows: the gripper’s length and gripper force should be moderate. Grippers that are too long will be penalized due to material costs and disturbances. Grippers that are too short will be penalized because they do not meet the feasibility of gripping conditions. Excessive gripper force will be penalized because it increases energy consumption and damages objects. Insufficient gripper force will be penalized because it cannot lift objects. Excessive friction will be penalized because it increases costs and wears the workpiece.

Constraint penalties: such as friction coefficient and gripper force constraints. An excessively low friction coefficient, combined with gripper force, can cause the frictional force to be less than gravity, preventing lifting and incurring a penalty.

These rewards are modeled using mathematical functions that combine multiple factors to determine whether a grab is successful.

### 3.5. Hyperparameter Settings and Training Tuning

#### 3.5.1. Hyperparameter Settings

In the experimental setup, this paper constructs a simulation environment for each parameter configuration and conducts independent interactive training within it [42]. A complete reinforcement learning training process is run once for each set of parameters: the agent continuously executes the closed-loop iteration of “decision-action-reward-update” in the environment, and the cumulative number of interaction steps is set to 40,000 time steps to ensure that the policy can reach a comparable convergence level under a fixed training budget. Training employs the Adam optimizer with a learning rate of 0.5%. During each update, 256 interactive data points are sampled from the empirical batch as a mini-batch for gradient updates, achieving a balance between convergence speed and training stability. To ensure fairness in comparisons across different parameter groups, this paper maintains a consistent network structure, reward function, training steps, and other hyperparameters, while varying only the parameter configurations to be evaluated. After training, the average reward, success rate, and optimal parameter values for each group are recorded for subsequent statistical analysis of performance and parameter-sensitivity comparisons.

#### 3.5.2. Training Optimization

**Figure 6:**
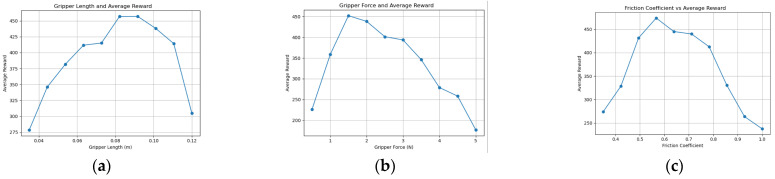
Parameters and reward training results of the robot gripper in Task 1. (**a**) Gripper length and reward training results. (**b**) Gripper force and reward training results. (**c**) Friction coefficient and reward training results.

**Figure 7:**
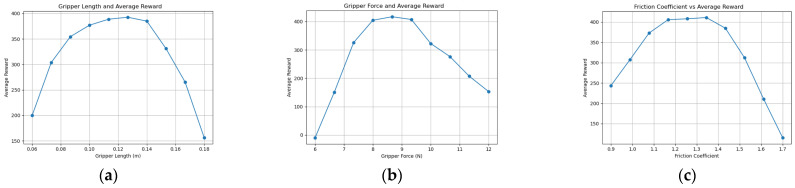
Parameters and reward training results of the robot gripper in Task 2. (**a**) Gripper length and reward training results. (**b**) Gripper force and reward training results. (**c**) Friction coefficient and reward training results.

**Figure 8:**
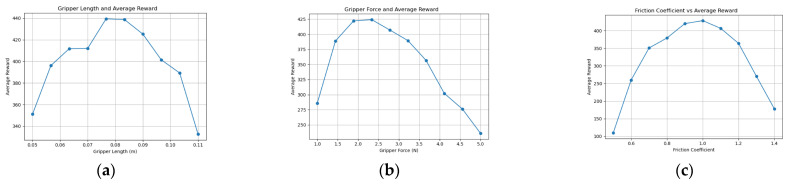
Parameters and reward training results of the robot gripper in Task 3. (**a**) Gripper length and reward training results. (**b**) Gripper force and reward training results. (**c**) Friction coefficient and reward training results.

With prior knowledge from the knowledge graph as input, the time step is set to 10,000, and the parameter range is divided into 10. The environment for this study was: Python 3.11.7, PyBullet 3.2.7, Windows 11, i9 13900HX processor. The training time for each set of parameters was approximately 2 h. In the experiment, the time step is set to 40,000, and the parameter range is divided into 30. The time for each set of parameters was approximately 20 h. When the parameter-scanning function is optimized for a given parameter, we use the robot end-effector knowledge graph and knowledge reasoning to calculate and fix the other parameters within a reasonable range to find the optimal value of the parameter. The optimal parameter values presented in this paper are determined through this process. The experimental training results of the KGPPO algorithm are shown in Figure 6, Figure 7 and Figure 8.

Through multiple explorations and continuous strategy updates, the algorithm discovered that when performing tasks 1, 2, and 3, the parameters of the two-finger translational gripper are concentrated around a specific value, and the average reward reaches its maximum. Based on this, optimal parameter values are determined, and an intelligent end-effector design is finally selected based on the parameter combination and task requirements.

### 3.6. Knowledge Graph Update

After obtaining the optimal end-effector parameter combination via reinforcement learning, we store the experimental results in the knowledge graph as structured knowledge to enable continuous accumulation and reuse of design knowledge, thereby supporting rapid solution generation and parameter initialization for subsequent similar tasks.

## 4. Experimental Results and Comparative Analysis

### 4.1. Example of a Knowledge Graph for Robot End-Effectors

**Figure 9:**
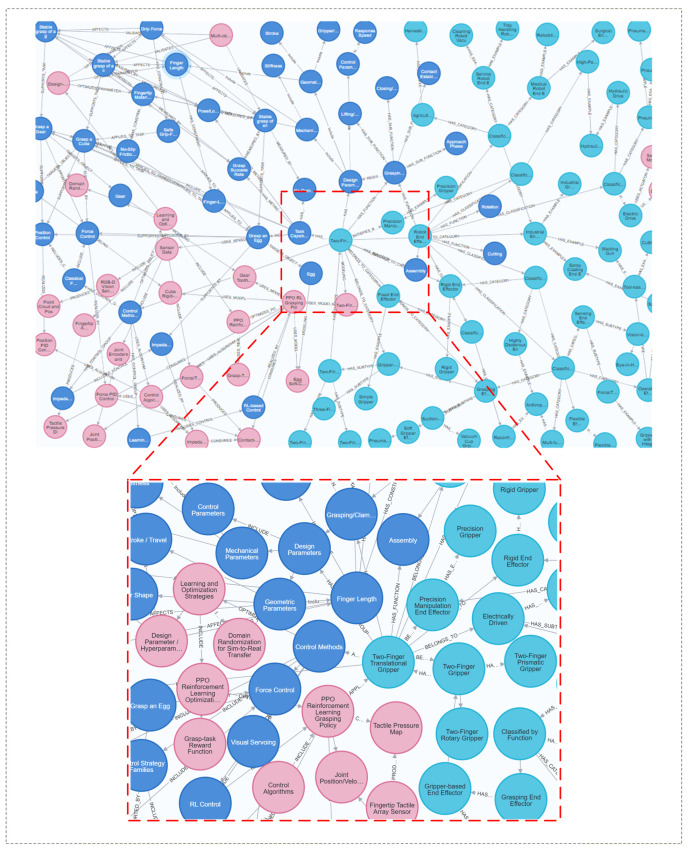
Visualization of the robot end-effector knowledge graph.

Given the current use of mainstream graph databases, the Neo4j library is selected for storing knowledge about robot end-effectors. This paper uses the py2neo program to access and call the Neo4j graph database. py2neo is a community-maintained third-party Python library that provides a simpler, more convenient way to perform Neo4j connection, data reading, writing, and querying operations within a Python environment, thereby improving the efficiency of graph database usage and integration. Following the construction process of the robot end-effectors knowledge graph in Section 3, a robot end-effectors knowledge graph is constructed, as shown in Figure 9.

### 4.2. Comparison of PPO Algorithms for Knowledge Graph Ablation

**Figure 10:**
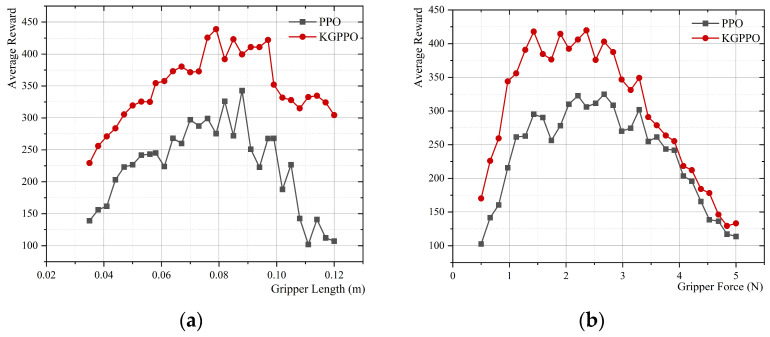
Parameters and rewards for grasping eggs, with and without knowledge graph guidance for the PPO algorithm. (**a**) Gripper length and reward. (**b**) Gripper force and reward.

**Figure 11:**
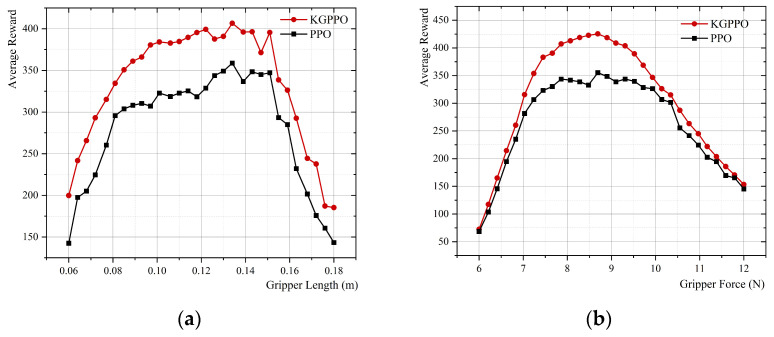
Parameters and rewards for grasping cubes, with and without knowledge graph guidance for the PPO algorithm. (**a**) Gripper length and reward. (**b**) Gripper force and reward.

**Figure 12:**
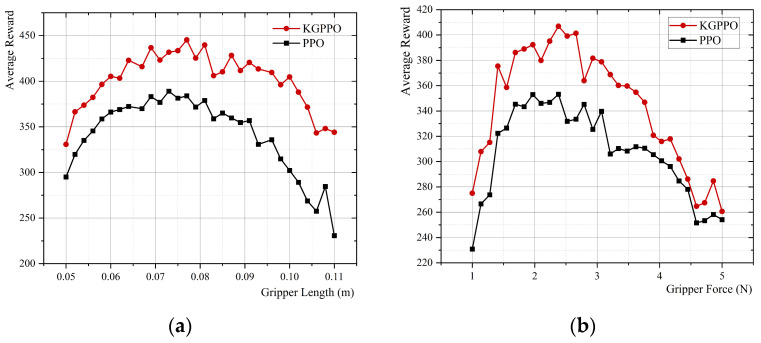
Parameters and rewards for grasping gears, with and without knowledge graph guidance for the PPO algorithm. (**a**) Gripper length and reward. (**b**) Gripper force and reward.

As shown in Figure 10, Figure 11 and Figure 12, without prior knowledge, each parameter set is run for 40,000 time steps in the environment, learning and exploring solely through the PPO algorithm, and the results are output. Then, the knowledge graph is integrated with the PPO algorithm for learning and exploration, and iterative feedback is performed; this is denoted as the KGPPO algorithm, and the results are output. This is used for comparison with the PPO algorithm in knowledge graph ablation.

The main results from data comparison and analysis are shown in Table 4, Table 5 and Table 6.

Analysis based on experimental results:

Performance Improvements: For the “average reward within the optimal length range,” compared to the PPO algorithm, the KGPPO algorithm improved rewards by 63.96% when grasping eggs, 19.47% when grasping cubes, and 11.11% when grasping gears. For the “average reward within the optimal gripper force range,” compared to the PPO algorithm, the KGPPO algorithm improved rewards by 43.09% when grasping eggs, 20.39% when grasping cubes, and 17.51% when grasping gears. This demonstrates that knowledge graph priors can effectively guide the strategy to find higher-quality feasible solutions, thereby increasing the upper limit of task performance.

Improved stability: The fluctuation range of the optimal gripper length was reduced by 72.97% when grasping eggs, by 58.33% when grasping cubes, and by 62.5% when grasping gears; the fluctuation range of the optimal gripper force was reduced by approximately 61.54% when grasping eggs, by 45.45% when grasping cubes, and by 66.67% when grasping gears, respectively. This indicates that prior knowledge reduced the ineffective exploration space, making the learning process more convergent and the results more stable.

Enhanced engineering usability: When grasping eggs, the optimal gripper force of KGPPO with a knowledge graph is 1.9 N, while the optimal gripper force of PPO without a knowledge graph is 2.4 N, a reduction of about 20.8%. While ensuring performance, it tends to use a smaller gripper force, which meets the safety requirements for grasping fragile objects. The gripper force remains almost constant when grasping the cube and the gears.

Therefore, knowledge graphs, as prior guidance, can significantly improve optimization quality and enhance the stability of the PPO algorithm, thereby making parameter optimization more efficient, reliable, and aligned with engineering design requirements.

### 4.3. Knowledge-Adaptive Design Output of the Robot End-Effectors

**Figure 13:**
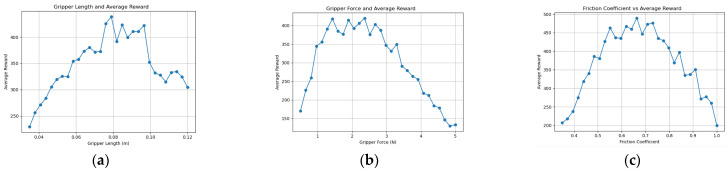
Relationship between various parameters of the robot gripper and the reward in Task 1. (**a**) Gripper length and reward. (**b**) Gripper force and reward. (**c**) Friction coefficient and reward.

**Figure 14:**
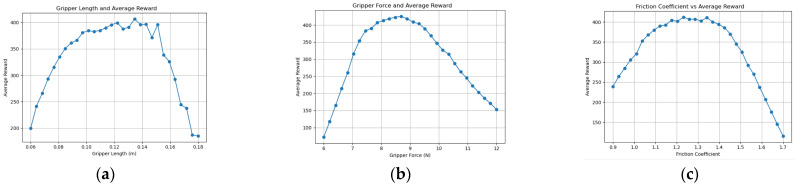
Relationship between various parameters of the robot gripper and the reward in Task 2. (**a**) Gripper length and reward. (**b**) Gripper force and reward. (**c**) Friction coefficient and reward.

**Figure 15:**
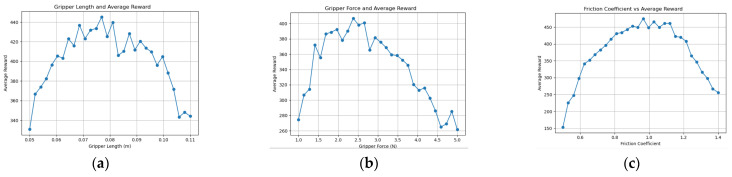
Relationship between various parameters of the robot gripper and the reward in Task 3. (**a**) Gripper length and reward. (**b**) Gripper force and reward. (**c**) Friction coefficient and reward.

Through comparative experiments using knowledge graph ablation, the optimization quality and stability of the knowledge graph-driven KGPPO algorithm are significantly enhanced, and this method is used for the adaptive design of robot end-effectors. Using the robot end-effectors knowledge graph as prior knowledge, each parameter set is executed for 40,000 time steps in the environment. The experimental results of implementing a two-finger translational end-effector using the KGPPO algorithm are shown in Figure 13, Figure 14 and Figure 15.

The data analysis above yielded the conclusions shown in Table 7. In Task 1, when the average reward is highest, the gripper length is 0.088 ± 0.01 m, the gripper force is 1.9 ± 0.5 N, and the coefficient of friction is 0.67 ± 0.05. In Task 2, when the average reward is highest, the gripper length is 0.137 ± 0.015 m, the gripper force is 8.7 ± 0.6 N, and the coefficient of friction is 1.3 ± 0.1. In Task 3, when the average reward is highest, the gripper length is 0.079 ± 0.003 m, the gripper force is 2.5 ± 0.2 N, and the coefficient of friction is 1.01 ± 0.11. Therefore, the parameters of the robot’s two-finger translational gripper can be set based on the gripper length, gripper force, and coefficient of friction given above.

### 4.4. Comparative Analysis of Multiple Algorithms Guided by Knowledge Graphs

This paper utilizes knowledge graphs to provide an initial solution, followed by multi-algorithm learning. In addition to the PPO algorithm, this paper introduces three other algorithms for comparison.

The Soft Actor-Critic (SAC) algorithm is an adaptive algorithm that balances stability and exploration and belongs to the family of reinforcement learning algorithms [43]. The SAC algorithm is a commonly used Actor-Critic method based on the Maximum Entropy framework in reinforcement learning and is suitable for continuous action spaces.

The Deep Deterministic Policy Gradient (DDPG) algorithm is a policy-dependent Actor-Critic algorithm for continuous action spaces that combines deterministic policy gradients with deep neural networks [44].

The Twin Delayed Deep Deterministic Policy Gradient (TD3) algorithm is an off-policy reinforcement learning algorithm for continuous action spaces that can be regarded as an improvement over DDPG [45].

A simulation environment is constructed by combining knowledge graphs. The above adaptive algorithms are then integrated into the robot end-effector knowledge graph, and adaptive optimization is performed. These algorithms are referred to as KGPPO, KGSAC, KGDDPG, and KGTD3. The primary hyperparameters of the four algorithms are shown in Table 8.

**Figure 16:**
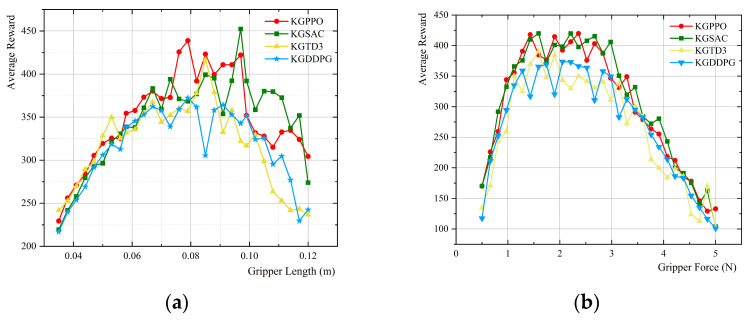
Algorithm comparison of gripper parameters and average reward for grasping eggs. (**a**) Algorithm comparison of gripper length and reward. (**b**) Algorithm comparison of gripper force and reward.

With prior knowledge as a guide, each set of parameters is executed for 40,000 time steps in the environment. After the knowledge graph participated in the adaptive design and iterative feedback, a multi-algorithm comparative learning exploration was carried out. The experimental output results are shown in Figure 16.

Based on the above data comparison and analysis, the conclusions are shown in Table 9.

Based on the analysis of experimental results and guidance from knowledge graphs, the following conclusions were drawn.

In terms of gripper length optimization, KGPPO offers the greatest advantage. The average reward of KGPPO across the optimal length range is 415.425, significantly higher than those of KGSAC, KGTD3, and KGDDPG, with corresponding improvements of approximately 9.93%, 13.06%, and 24.15%, respectively. Furthermore, KGPPO provides an optimal length of 0.088 ± 0.010 m with the smallest variance, indicating more stable convergence and more reproducible results; while KGSAC and KGDDPG exhibit larger length fluctuations, indicating greater uncertainty.

In terms of gripper force optimization, KGPPO is almost on par with KGSAC, but significantly better than KGTD3 and KGDDPG. The average reward of KGPPO for the optimal gripper force range is 397.231, which is basically the same as KGSAC; however, it is about 12.02% and 19.44% higher than KGTD3 and KGDDPG, respectively.

The gripper force of KGPPO is more “conservative and stable,” better suited to grasping fragile objects. The gripper force of KGPPO is 1.9 ± 0.5 N, exhibiting not only a smaller mean but also minimal fluctuation. This means that while ensuring task performance, KGPPO is more likely to achieve design parameter solutions with lower damage risk and greater robustness.

In summary, under the guidance of prior knowledge graphs, all four algorithms can converge to a reasonable range of optimal design parameters. However, the KGPPO algorithm offers the highest and most stable reward in “length optimization,” while the KGSAC algorithm’s average reward is almost equal to that of the KGPPO algorithm in “gripper force optimization,” but its volatility is greater. The KGTD3 and KGDDPG algorithms generally have lower rewards and greater parameter fluctuations. KGPPO demonstrates the greatest overall advantage across both parameter optimization methods; therefore, given all factors, the KGPPO algorithm is the preferred choice for knowledge-guided design optimization.

### 4.5. Optimal Parameter Evaluation and Convergence Analysis

**Figure 17:**
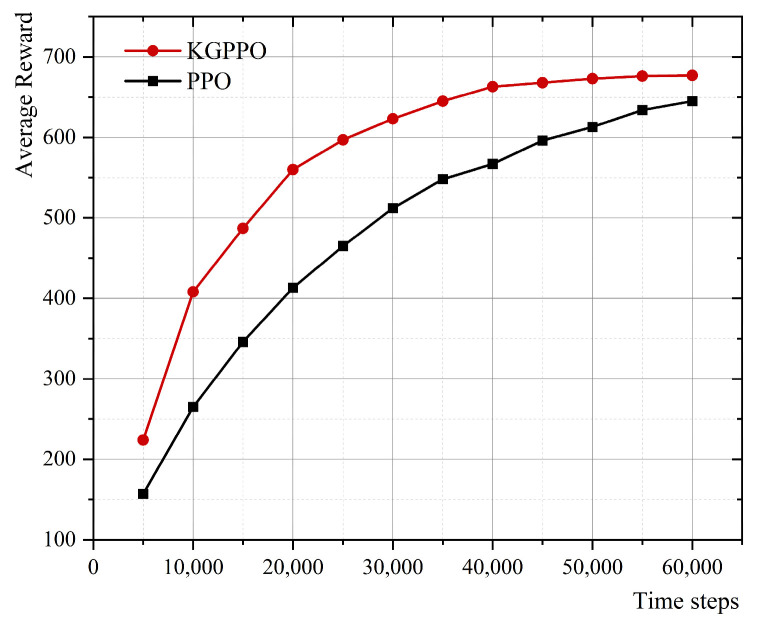
The relationship between time steps and average reward under the optimal parameter combination for grasping eggs.

The above method obtains the optimal value of a certain parameter by fixing the remaining parameters. After obtaining all optimal design parameters, they are fixed and applied during the grasp eggs process to verify the learning efficiency and convergence performance of the proposed method. Specifically, experiments were conducted using the KGPPO and PPO algorithms under the same environmental conditions. The learning process of the algorithms was analyzed by recording changes over time steps and average rewards during training. As the number of time steps increases, the robot end-effector gradually optimizes its strategy through continuous interaction with the environment, and the average reward increases accordingly and eventually stabilizes. When the average reward no longer shows significant growth and remains in a stable range, the corresponding time step is considered the convergence step number. By comparing the convergence curves and convergence steps of KGPPO and PPO during learning, the role of the knowledge-guidance mechanism in reinforcement learning and its impact on improving task execution efficiency and learning stability can be further evaluated. The results are shown in Figure 17.

The results show that the average reward of the KGPPO algorithm converges after 40,000 time steps. After 40,000 iterations, the average reward from grasping eggs is significantly higher than that with a single optimal parameter, thereby verifying the rationality of the proposed method for determining the optimal design parameters of the robot end-effector.

### 4.6. Subsection

**Figure 18:**
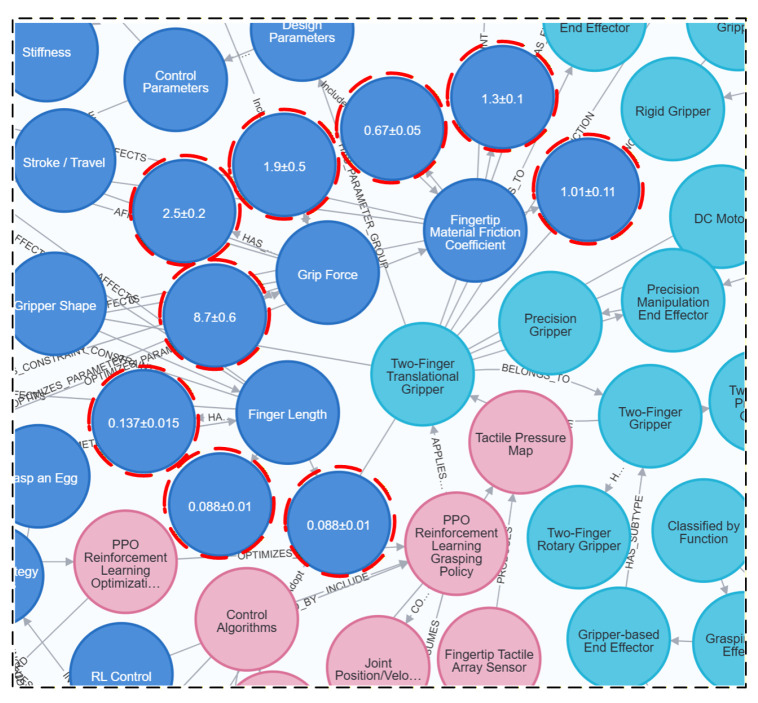
Display of updated knowledge graph results.

After performing three different tasks, the design parameters of the two-finger translational gripper will be finalized and entered into the completed robot end-effectors knowledge graph for updating. The updated results are shown in Figure 18.

## 5. Conclusions and Future Work

This paper proposes a knowledge-adaptive design method based on “knowledge graph, environmental perception, and adaptive algorithm” for robot end-effectors. The knowledge of the robot end-effectors domain is structured, modeled, and stored as a knowledge graph, and is deeply embedded in it during the design process. By constructing a simulation environment and treating key design parameters as environmental variables, the algorithm can continuously perceive and respond to environmental feedback to find better parameter values during robot end-effectors task execution, significantly improving design quality and result accuracy. The experimental results are fed back to the knowledge graph to update the design knowledge. Specific research results are as follows.

A knowledge graph for robot end-effectors was creatively constructed around three levels: “category, capability parameters, and specific implementation.” This enabled the storage, visualization, and updating of knowledge about the robot end-effectors. An ablation experiment was conducted on the knowledge graph. Over 40,000 time steps, compared with no prior knowledge input, the KGPPO algorithm improved the average reward for length and gripper force by 63.96% and 43.09%, respectively. The optimal values of the main design parameters of the two-finger translational gripper were obtained through experiments on three tasks. In multi-algorithm comparison experiments, guided by knowledge graph priors, KGPPO improved the “average reward for the optimal length range” metric by approximately 9.93% compared to KGSAC, approximately 13.06% compared to KGTD3, and approximately 24.15% compared to KGDDPG. In the “average reward for the optimal gripper force range,” KGPPO was roughly on par with KGSAC, but improved by approximately 12.02% and 19.44% compared to KGTD3 and KGDDPG, respectively. Furthermore, the KGPPO’s output for optimal parameters was more concentrated. This indicates that KGPPO has better convergence stability and repeatability under prior constraints, making it a more suitable optimization algorithm for the method presented in this paper.

Future work can be carried out in the following areas. Firstly, the mechanisms for scaling and reasoning of knowledge graphs. This involves introducing automated knowledge-extraction and ontology-evolution methods and integrating literature, standards, specifications, product manuals, and experimental data. Knowledge-based reasoning improves convergence speed, enabling stable results with fewer iterations. Secondly, experimental verification in real-world scenarios. Building on the method verification in the simulation environment, the next step of the research will be to conduct further experimental studies on a real robot platform, quantitatively evaluate and compare the design results and the effects of strategy execution, and verify the method’s feasibility and stability in real physical environments.

## Figures and Tables

**Table 1:** Main design parameters of the robot end-effectors.

Parameter Type	Specific Parameters	Meaning
Geometric parameters	Finger length	Length of the finger from base to fingertip
	Finger width	The width of the fingers affects the contact area
	Finger thickness	The thickness of the finger affects its structural strength
	Finger shape	Including rectangles, circles, and shapes with outlines
Motion parameters	Opening and closing speed	Finger movement speed
	Acceleration	The acceleration of finger movement affects dynamic performance
	Stroke	The distance the fingers move from fully closed to fully open
Force parameters	Gripper force	The gripping force that the claw can apply
	Coefficient of friction	The coefficient of friction between a finger and an object
Control parameters	Control method	Position control, force control, or a combination of both
	Sensor type	Such as position, force, and tactile sensors
	Driving method	Electric, pneumatic, or hydraulic
Other parameters	Operating temperature	Normal operating temperature range of the hand
	Weight	The weight of the claw itself
	Power failure protection	The state of the gripper when the power is off
	Noise level	Noise during operation

**Table 2:** Performance evaluation of knowledge extraction.

Task	Precision	Recall	F1-Score
Entity Extraction	0.91	0.88	0.89
Relation Extraction	0.88	0.85	0.86

**Table 3:** Fixed reward values.

Fixed Rewards	Numerical Values	Describe
Geometric feasibility reward	+0.6	The gripper can wrap around an object and earn a reward.
Contact reward	+0.5	A reward is given for touching the target object with two fingers.
Alignment reward	+0.4	A reward is given for aligning with the target object.
Lifting reward	+0.7	A reward is given for lifting the target object to a certain threshold.
Placement reward	+0.3	A reward is given for placing the target object in the designated location.

**Table 4:** Comparison of algorithms with and without knowledge graph prior guidance for grasping eggs.

	KGPPO	PPO
Optimal gripper length	0.088 ± 0.01 m	0.087 ± 0.037 m
Average reward of the optimal range of gripper length	415.425	253.367
Optimal gripper force	1.9 ± 0.5 N	2.4 ± 1.3 N
Average reward of the optimal range of gripper force	397.231	277.613

**Table 5:** Comparison of algorithms with and without knowledge graph prior guidance for grasping cubes.

	KGPPO	PPO
Optimal gripper length	0.137 ± 0.015 m	0.119 ± 0.036 m
Average reward of the optimal range of gripper length	386.642	323.627
Optimal gripper	8.7 ± 0.6 N	8.6 ± 1.1 N
Average reward of the optimal range of gripper force	412.574	342.684

**Table 6:** Comparison of algorithms with and without knowledge graph prior guidance for grasping gears.

	KGPPO	PPO
Optimal gripper length	0.079 ± 0.003 m	0.075 ± 0.008 m
Average reward of the optimal range of gripper length	418.265	376.435
Optimal gripper force	2.5 ± 0.2 N	2.4 ± 0.6 N
Average reward of the optimal range of gripper force	396.243	337.214

**Table 7:** Output results of the knowledge-adaptive design.

	Gripper Length (m)	Gripper Force (N)	Friction Coefficient
Execute Task 1	0.088 ± 0.01	1.9 ± 0.5	0.67 ± 0.05
Execute Task 2	0.137 ± 0.015	8.7 ± 0.6	1.3 ± 0.1
Execute Task 3	0.079 ± 0.003	2.5 ± 0.2	1.01 ± 0.11

**Table 8:** Main hyperparameters of each algorithm.

Hyperparameter	KGPPO	KGSAC	KGTD3	KGDDPG
Learning rate	$3\times 10^{-4}$	$3\times 10^{-4}$	$3\times 10^{-4}$	$3\times 10^{-4}$
Batch size	256	256	256	256
Discount rate	0.99	0.99	0.99	0.99
Shear range (ε)	0.2	-	-	-
Entropy coefficient	-	0.1	-	-
Time step	40,000	40,000	40,000	40,000
Sample step	200	200	200	200
episode	10	10	10	10
Explore noise	-	-	0.1	0.1

**Table 9:** Comparison of algorithms guided by knowledge graph priors.

	KGPPO	KGSAC	KGTD3	KGDDPG
Optimal gripper length for grasping eggs	0.088 ± 0.010 m	0.090 ± 0.028 m	0.079 ± 0.017 m	0.080 ± 0.028 m
Average reward of the optimal range of gripper length	415.425	377.892	367.432	334.628
Optimal gripper force for grasping eggs	1.9 ± 0.5 N	2.2 ± 1.0 N	2.2 ± 1.1 N	2.25 ± 1.35 N
Average reward of the optimal range of gripper force	397.231	397.162	354.617	332.586

## Data Availability

Data are contained within the article.

## Abbreviations

The following abbreviations are used in this manuscript:

PPO	Proximal Policy Optimization
KG	Knowledge Graph
KGPPO	Knowledge Graph Embedding Proximal Policy Optimization
BERT	Bidirectional Encoder Representations from Transformers
CRF	Conditional Random Field
KGSAC	Knowledge Graph Embedding Soft Actor-Critic
KGDDPG	Knowledge Graph Embedding Deep Deterministic Policy Gradient
KGTD3	Knowledge Graph Embedding Twin Delayed Deep Deterministic Policy Gradient
